# Association of occupational burnout and perceived healthcare service quality with medical disputes among physicians and medical technicians: a cross-sectional study in China

**DOI:** 10.1186/s12913-026-15142-5

**Published:** 2026-07-13

**Authors:** Ziyang Liu, Jie Wang, Wenhui Zou, Zijian Qi, Liang Shen

**Affiliations:** 1 School of Management, Xuzhou Medical University, Xuzhou, China; 2 School of Public Health, Xuzhou Medical University, Xuzhou, China; 3 Office for Professional Conduct, Xuzhou Children’s Hospital, Xuzhou, China; 4 Suining People’s Hospital, Xuzhou, China; 5 Department of Infection Control, Wujin Hospital Affiliated with Jiangsu University, Changzhou, China; 6 Department of Infection Control, The Wujin Clinical college of Xuzhou Medical University, Changzhou, China

**Keywords:** Medical disputes, Influencing factors, Occupational burnout, Healthcare service quality

## Abstract

**Purpose:**

This study aimed to investigate the associations between occupational burnout, perceived healthcare service quality, and medical disputes among physicians and medical technicians, and to identify factors associated with medical disputes from their perspective.

**Methods:**

A cross-sectional study was conducted among 1570 participants in Xuzhou, China, between October and November 2025. Hierarchical logistic regression was used to assess the associations of perceived healthcare service quality and occupational burnout with medical disputes.

**Results:**

Among 1570 respondents, 283 (18.0%) reported experiencing at least one medical dispute in the past year. Higher perceived healthcare service quality (OR = 0.980, 95% CI: 0.963, 0.997) was associated with lower odds of medical disputes. In contrast, higher occupational burnout (OR = 1.059, 95% CI: 1.037, 1.082), male gender (OR = 2.459, 95% CI: 1.802, 3.357), working 40–55 (OR = 2.006, 95% CI: 1.112, 3.618) or over 55 h per week (OR = 2.408, 95% CI: 1.307, 4.437), seeing 20–30 (OR = 1.725, 95% CI: 1.184, 2.513), 30–40 (OR = 1.734, 95% CI: 1.037, 2.899) or more than 40 patients daily (OR = 1.899, 95% CI: 1.305, 2.765), and turnover intention (OR = 1.741, 95% CI: 1.233, 2.459) were associated with higher odds of disputes.

**Conclusions:**

Higher burnout levels, longer working hours, and greater daily patient volumes were associated with an increased likelihood of reporting medical disputes, whereas higher perceived healthcare service quality was associated with a decreased likelihood of reporting such experiences. Healthcare organizations may consider implementing interventions that target workload management, burnout reduction, and communication improvement.

**Supplementary Information:**

The online version contains supplementary material available at https://doi.org/10.1186/s12913-026-15142-5.

## Introduction

Medical disputes represent one of the most pervasive and complex challenges in the contemporary global healthcare landscape. Broadly defined as conflicts arising from actions or errors by healthcare professionals that deviate from accepted standards and cause patient harm, these disputes span civil, administrative, and criminal domains [1]. As healthcare systems expand and patient awareness of rights increases, the prevalence of medical disputes has become a critical indicator of the quality and stability of healthcare institutions [2], underscoring the delicate balance between clinical necessity and patient satisfaction.

In recent years, the frequency, severity, and consequences of medical disputes have intensified globally, driven by economic growth, greater health literacy, and an increasing emphasis on individual rights [3, 4]. International data illustrate the growing strain. In the United States, approximately 7.4% of physicians face malpractice lawsuits annually, with 1.6% resulting in indemnity payments [5]. In Germany, among 108,000 insured physicians, 4.2% were confronted with malpractice claims, and 10% of these cases proceeded to court [6]. The situation is especially acute in parts of Asia. In South Korea, the number of malpractice lawsuits increased fourteenfold between 1989 and 2006, with more than 60% of physicians reporting involvement in disputes [7]. In China, data from the National Health Commission show that in 2015, medical institutions recorded 73 million outpatient visits, among which approximately 70,000 cases led to disputes—a substantial rise from 17,243 cases in 2010. These figures do more than quantify administrative burdens; they reflect a deepening erosion of mutual trust. Alarmingly, studies indicate that 42.2% to 83.3% of healthcare workers have experienced workplace violence, suggesting an increasingly adversarial care environment [8, 9]. Such disputes inflict substantial psychological distress, financial burden, and reputational risk on both providers and patients, and may foster defensive medical practices that ultimately undermine patient safety [10].

Addressing this growing crisis requires an understanding of how academic and practical perspectives on medical disputes have evolved. Early scholarship largely emphasized legal and institutional frameworks, focusing on post hoc handling mechanisms such as medico-legal regulations, insurance compensation, and administrative arbitration processes [11, 12]. While these studies established a foundation for dispute resolution, they provided relatively limited evidence regarding strategies for dispute prevention. Later research shifted toward patient-related and structural determinants, identifying unrealistic expectations, limited health literacy, and systemic inefficiencies—such as overcrowding and insufficient consultation time—as dominant drivers [13]. Much of this work, however, treats healthcare providers as passive actors within the system, overlooking the human, relational, and experiential dimensions of care [10, 14].

Despite these advances, important limitations persist in the existing literature. Although communication quality and patient-centered care have been widely acknowledged as relevant to medical disputes, most studies address these factors in isolation and at a relatively superficial level, without systematically examining the potential mechanisms that may underlie healthcare providers’ behaviors during clinical interactions [15]. In particular, research has tended to conceptualize healthcare professionals primarily as functional actors within organizational and regulatory systems, with limited attention to their psychological states, emotional labor, and adaptive responses to sustained work stress [16]. Moreover, empirical evidence linking provider-related behavioral or psychological factors to the occurrence of medical disputes remains fragmented, and few studies have integrated these dimensions into comprehensive analytical frameworks. As a result, the relational and experiential processes through which disputes emerge in routine clinical practice remain insufficiently understood.

A notable gap persists that limited attention has been given to internal psychological and behavioral factors among healthcare professionals that may predispose to or intensify disputes. Healthcare delivery is inherently relational. Beyond technical competence, the effectiveness of communication, empathy, and responsiveness critically shape patient perceptions [17–19]. Under conditions of sustained occupational stress, healthcare professionals experiencing burnout may become emotionally exhausted, detached, or less responsive during clinical encounters. Burnout has been associated with emotional exhaustion, reduced empathy, and communication difficulties during clinical encounters [20, 21]. In turn, poor communication and reduced service quality may contribute to unmet expectations, dissatisfaction, and mistrust among patients and their families, thereby increasing the likelihood that routine clinical conflicts escalate into formal medical disputes [22]. From this perspective, burnout may not only influence disputes directly but also indirectly, through its impact on interpersonal interactions and perceived healthcare service quality.

Healthcare service quality may represent an important factor associated with the relationship between healthcare professionals’ psychological well-being and medical dispute experiences. Healthcare service quality encompasses not only clinical precision but also timeliness, safety, interpersonal interactions, and overall patient experience [23]. Variations in perceived service quality reflect institutional capacity, workload distribution, and professional competencies. When medical staff perceive that systemic constraints compromise their ability to deliver high-quality care, dissatisfaction may arise on both sides, heightening the likelihood of disputes [24]. Therefore, healthcare service quality may represent an important dimension linking healthcare professionals’ working experiences with medical dispute experiences. Yet few studies have jointly examined healthcare service quality and provider burnout to understand their combined influence on dispute incidence.

Against this backdrop, the present study investigates medical disputes from the perspective of healthcare professionals. Specifically, we evaluate how demographic and occupational characteristics, burnout levels, and perceived healthcare service quality are associated with medical dispute experiences. By examining the relationships among healthcare professionals’ psychological characteristics, perceived healthcare service quality, and medical dispute experiences, this study aims to improve understanding of how these factors may be associated with disputes within the doctor–patient relationship (DPR). The findings aim to provide evidence to inform future research and organizational strategies aimed at reducing medical disputes.

## Methods

### Study design and participants

A cross-sectional study was conducted between October and November 2025. Participants were recruited through a multistage sampling procedure. The study was conducted in Xuzhou, Jiangsu Province, located in eastern China.

Prior to the formal survey, the research team carried out preliminary field investigations, including face-to-face communications and discussion meetings with administrative leaders from public hospitals and community healthcare institutions. These investigations enabled the researchers to gain a more nuanced understanding of staffing structures and professional responsibilities in grassroots healthcare settings. It was observed that, particularly in certain community and township healthcare institutions, clinical responsibilities may partially overlap across different professional groups.

In the first stage, all administrative districts and counties within Xuzhou were identified as sampling units. In the second stage, within each district or county, one tertiary public hospital and two community hospitals were randomly selected to ensure coverage of different healthcare settings. In the third stage, physicians and medical technicians employed in these institutions were invited to participate through an online questionnaire survey. The survey link was distributed through institutional coordinators, and participants completed the questionnaire electronically. All responses were collected through a secure online survey platform. Given the study objective, this study focused on healthcare professionals directly involved in clinical diagnosis, treatment, and related medical technical services. Therefore, nurses, administrative staff, and support staff were excluded from the study.

A total of 1738 participants provided informed consent and completed the questionnaire, including 1298 physicians and 440 medical technicians. After excluding 118 physicians and 50 medical technicians with missing data on key variables, 1570 respondents (1180 physicians and 390 medical technicians) were retained for the final analysis. This sampling process ensured adequate representativeness of physicians and medical technicians across different hospital levels and administrative districts within Xuzhou.

### Research instrument

The questionnaire was developed through a multi-stage process that included a literature review, expert consultation, and pilot testing (the complete questionnaire is provided in Supplementary 1). Systematic searches were conducted in PubMed, Web of Science, MEDLINE, China National Knowledge Infrastructure (CNKI), and Wanfang databases using keywords such as “medical disputes,” “medical accidents,” “medical malpractice,” and “influencing factors.” Relevant determinants of medical disputes were identified, synthesized, and categorized to inform the initial draft of the questionnaire. Two rounds of Delphi expert consultations were subsequently held with five specialists in hospital management, social medicine and health service management, and hospital disciplinary inspection. Experts assessed each item for relevance, feasibility, and sensitivity, and feedback from each round was used to iteratively refine the questionnaire, yielding a satisfactory level of expert consensus. Based on the pilot results, minor revisions were made to ensure that the final instrument demonstrated acceptable reliability, stability, and applicability.

Prior to the survey, all investigators received standardized training covering the research design, objectives, methodology, and detailed questionnaire content to ensure a thorough understanding of the study background and overall framework. Specific aspects, including questionnaire administration procedures, implementation requirements, timelines, and expected workload, were comprehensively explained. This process enabled investigators to become fully familiar with the questionnaire structure, completion procedures, and critical points requiring attention. In accordance with formal survey protocols, a pilot survey was conducted to allow investigators to rehearse the entire data collection process. Issues identified during this simulation were subsequently addressed through group discussions and targeted clarifications. These refinements focused on organizational and management arrangements, guidance and supervision mechanisms, review and quality inspection procedures, as well as systems for summarizing and sharing findings.

### Measurement

#### Definition of medical dispute

In the questionnaire, participants were presented with the following uniform definition: “All conflicts between medical practitioners and patients (and their families) related to diagnosis and treatment. These primarily include verbal abuse, minor physical altercations (e.g. shoving), online harassment, personal attacks, and obstruction of medical service (e.g. blocking hospital entrances).” Participants were then asked whether they had experienced such an incident at least once in the past 12 months (yes/no).

In the present study, the term “medical dispute” was intentionally defined broadly to capture conflict-related incidents occurring in routine clinical practice, including early-stage interpersonal conflicts that may not escalate to formal legal or administrative malpractice claims [1]. This operational definition reflects the practical healthcare environment in China, where dispute-related events often manifest as verbal aggression, service obstruction, or other forms of workplace conflict. Accordingly, the outcome variable should be interpreted as participants’ exposure to healthcare conflict incidents rather than exclusively as formal malpractice disputes.

#### Occupational burnout

Burnout was assessed with the 16-item Oldenburg Burnout Inventory (OLBI) [25, 26], which comprises two subscales: exhaustion (EX) and disengagement (DEM). Each item is rated on a 4-point Likert scale, with a total score ranging from 16 to 64; higher scores indicate more severe burnout. The OLBI has shown good reliability and construct validity in previous studies and has been widely applied and validated among Chinese healthcare professionals [27]. In the present study, the overall Cronbach’s α coefficient was 0.862, indicating satisfactory internal consistency.

#### Perceived healthcare service quality

The perceived healthcare service quality was evaluated using the Chirurgisches Qualitätssiegel (CQS) [21]. The CQS comprises 13 items across three dimensions: Psychosocial Care, Diagnostic Methods, and Quality Assurance. The Psychosocial Care dimension reflects the psychological and emotional support provided by healthcare personnel to patients and their families. Diagnostic Methods assess the professional competence and technical proficiency of healthcare providers, while Quality Assurance captures factors that ensure the overall integrity of the medical process. Participants rated each item on a five-point Likert scale ranging from 1 (“Very Poor”) to 5 (“Very Good”), with higher scores representing better perceived healthcare service quality [28]. In the present study, the overall Cronbach’s α coefficient was 0.942, indicating excellent internal consistency.

#### Key covariates measurement

Mental health status, communication status, and turnover intention were assessed using single-item self-reported measures. Participants were asked: “How would you rate your current mental health status?”, “How would you rate your communication with patients and their families?”, and “Do you have turnover intention (intention to resign or change profession)?” Mental health status was rated on a 5-point Likert scale ranging from 1 (“very poor”) to 5 (“very good”). Communication status was rated on a 3-point scale ranging from 1 (“poor”) to 3 (“good”). Turnover intention was assessed as a binary variable (“yes” or “no”). Higher scores indicated better mental health and communication status, while turnover intention was coded as 1 = yes and 0 = no.

In addition, Professional title was categorized objectively according to officially recognized professional rank as none, junior, intermediate, and associate senior/senior. Income level was assessed using participants’ subjective evaluation of their income status and categorized as low, general, or high.

#### Covariates

Covariates were selected based on prior literature and potential relevance to medical disputes among physicians. The following variables were included: gender, age, education level, marital status, professional title, working hours per week, daily patient volume, income level, undertaking teaching duties, performing administrative duties, medical liability insurance, mental health status, communication status, and turnover intention.

### Data analysis

Categorical variables were summarized as frequencies and percentages (%), and group differences were examined using chi-square or fisher’s tests. Continuous variables were reported as mean ± standard deviation (SD) and compared between groups using two-sample t tests. Multivariable logistic regression analyses were conducted to examine the associations between occupational burnout, perceived healthcare service quality, and medical disputes. Candidate covariates were selected based on prior literature and bivariate analyses, with variables showing *p* < 0.15 in bivariate analyses considered for model inclusion. In addition, several theoretically relevant variables identified from previous studies were retained regardless of their bivariate statistical significance to reduce the risk of residual confounding. Prior to regression analyses, multicollinearity among independent variables was assessed using variance inflation factors (VIFs) and tolerance statistics. Model (1) solely included occupational burnout and healthcare service quality, while Model (2) also encompassed significant covariates identified in the bivariate analysis. Statistical analyses were conducted using R software (version 4.5.2) and SPSS software (version 26.0), with a significance level of *p* < 0.05 (two-sided) considered statistically significant.

## Results

### Participants characteristics by groups

Table 1 presents the participants’ characteristics, levels of occupational burnout, and healthcare service quality across groups. Among the 1570 participants surveyed, 283 (18.0%) reported having experienced at least one medical dispute within the past year. The majority of participants were female (1132, 72.1%), and most (1397, 88.9%) were under 50 years of age. The predominant educational level was a bachelor’s degree, accounting for 876 participants (55.8%).

**Table 1:** Socio-demographic characteristics and key variables of participants

Characteristic	Total	Medical disputes		Statistics	
	(*N* = 1570)	Yes (*n* = 283)	No (*n* = 1287)	χ^2^/t	*P*
Gender (n, %)				41.579	< 0.001***
Male	438(27.9)	123(43.5)	315(24.5)		
Female	1132(72.1)	160(56.5)	972(75.5)		
Age (n, %)				4.518	0.333
≤ 30	504(32.1)	81(28.6)	423(32.9)		
31–40	559(35.6)	109(38.5)	450(35.0)		
41–50	334(21.3)	58(20.5)	276(21.4)		
≥ 51	173(11.0)	35(12.4)	138(10.7)		
Marital status (n, %)				0.422	0.81
Single	432(27.5)	75(26.5)	357(27.7)		
Married	1138(72.5)	208(73.5)	930(72.3)		
Education level (n, %)				20.647	0.001**
Below bachelor	664(42.3)	89(31.5)	575(44.7)		
Bachelor degree	876(55.8)	185(65.4)	691(53.7)		
Master or above	30(1.9)	9(3.2)	21(1.6)		
Professional title (n, %)				11.908	0.018*
No	238(15.2)	31(11.0)	207(16.1)		
Junior	597(38.0)	96(33.9)	501(38.9)		
Intermediate	492(31.3)	99(35.0)	393(30.5)		
Senior	243(15.5)	57(20.1)	186(14.5)		
Working hours per week (n, %)				43.814	< 0.001***
<40	217(13.8)	15(5.3)	202(15.7)		
40–55	898(57.2)	146(51.6)	752(58.4)		
>55	455(29.0)	122(43.1)	333(25.9)		
Daily patient volume (n, %)				27.208	< 0.001***
<20	684(43.6)	85(30.0)	599(46.5)		
20–30	379(24.1)	79(27.9)	300(23.3)		
30–40	148(9.4)	32(11.3)	116(9.0)		
>40	359(22.9)	87(30.7)	272(21.1)		
Income level (n, %)				1.618	0.47
High	26(1.7)	4(1.4)	22(1.7)		
moderate	827(52.7)	140(49.5)	687(53.4)		
Low	717(45.7)	139(49.1)	578(44.9)		
Undertake teaching duties (n, %)				6.525	0.011*
Yes	176(11.2)	44(15.5)	132(10.3)		
No	1394(88.8)	239(84.5)	1155(89.7)		
Perform administrative duties (n, %)				8.878	0.003**
Yes	271(17.3)	66(23.3)	205(15.9)		
No	1299(82.7)	217(76.7)	1082(84.1)		
Medical liability insurance (n, %)				2.644	0.104
Yes	1180(75.2)	202(71.4)	978(76.0)		
No	390(24.8)	81(28.6)	309(24.0)		
Mental health status (n, %)				107.901	< 0.001***
Very good	545(34.7)	50(17.7)	495(38.5)		
Good	472(30.1)	67(23.7)	405(31.5)		
Neutral	482(30.7)	131(46.3)	351(27.3)		
Bad	57(3.6)	28(9.9)	29(2.3)		
Very bad	14(0.9)	7(2.5)	7(0.5)		
Communication status (n, %)				25.362	< 0.001***
Good	1431(91.1)	235(83.0)	1196(92.9)		
Neutral	137(8.7)	47(16.6)	90(7.0)		
Bad	2(0.1)	1(0.4)	1(0.1)		
Turnover intention (n, %)				93.686	< 0.001***
Yes	390(24.8)	134(47.3)	256(19.9)		
No	1180(75.2)	149(52.7)	1031(80.1)		
Occupational burnout (mean ± SD)	37.13 ± 7.49	42.21 ± 8.404	36.02 ± 6.78	-13.283	< 0.001***
Healthcare service quality (mean ± SD)	55.84 ± 8.16	53.65 ± 8.62	56.32 ± 7.98	5.904	< 0.001***

Notes: *P < 0.05, **P < 0.01, ***P < 0.001

Variables associated with medical disputes at *p* < 0.15 in univariate analyses were considered candidates for multivariable logistic regression. These included gender, education level, professional title, working hours per week, daily patient volume, undertaking teaching duties, performing administrative duties, mental health status, communication status, turnover intention, medical liability insurance, occupational burnout, and perceived healthcare service quality. In addition, income level was included in the multivariable model based on prior literature and their potential roles as confounding factors, regardless of their univariate significance.

Prior to multivariable regression analysis, multicollinearity among independent variables was assessed using VIFs. As shown in Table 2, the results showed that all VIF values were below 5, indicating no evidence of substantial multicollinearity among the predictors included in the regression models.

**Table 2:** VIF analysis of independent variables

Variable	VIF	Tolerance(1/VIF)
Turnover intention	1.348	0.742
Mental health status	1.266	0.790
Occupational burnout	1.529	0.654

Notes: VIF = Variance inflation factor

### Factor influencing medical disputes through hierarchical regression analysis

Table 3 presents the factors associated with medical disputes identified through hierarchical logistic regression analysis. In Model 1, when only occupational burnout and perceived healthcare service quality were included, the results showed that higher occupational burnout was associated with higher odds of medical disputes (OR = 1.095, 95% CI: 1.077, 1.113), whereas higher perceived healthcare service quality was associated with lower odds of medical disputes (OR = 0.974, 95% CI: 0.959, 0.989). In Model 2, additional covariates were incorporated into the analysis. The findings indicated that, Higher perceived healthcare service quality (OR = 0.980, 95% CI: 0.963, 0.997) was associated with lower odds of medical disputes. In contrast, higher occupational burnout (OR = 1.059, 95% CI: 1.037, 1.082), male gender (OR = 2.459, 95% CI: 1.802, 3.357), working 40–55 (OR = 2.006, 95% CI: 1.112, 3.618) or over 55 h per week (OR = 2.408, 95% CI: 1.307, 4.437), seeing 20–30 (OR = 1.725, 95% CI: 1.184, 2.513), 30–40 (OR = 1.734, 95% CI: 1.037, 2.899) or more than 40 patients daily (OR = 1.899, 95% CI: 1.305, 2.765), and turnover intention (OR = 1.741, 95% CI: 1.233, 2.459) were associated with higher odds of disputes.

**Table 3:** Influencing factors of multivariate regression of medical disputes model

Medical disputes	Model 1	Model 2
Occupational burnout	1.095(1.077,1.113) ***	1.059(1.037,1.082) ***
Healthcare service quality	0.974(0.959,0.989) ***	0.980(0.963,0.997) *
Gender		
Female		Ref.
Male		2.459(1.802,3.357) ***
Working hours per week		
<40		Ref.
40–55		2.006(1.112,3.618) *
>55		2.408(1.307,4.437) **
Daily patient volume		
<20		Ref.
20–30		1.725 (1.184,2.513) **
30–40		1.734(1.037,2.899) *
>40		1.899(1.305,2.765) ***
Turnover intention		
No		Ref.
Yes		1.741(1.233,2.459) **

Notes: *P < 0.05, **P < 0.01, ***P < 0.001. Ref. = Reference

Each one-point increase in the OLBI burnout score was associated with approximately 5.9% higher odds of medical disputes. Correspondingly, a 5-point increase in burnout score was associated with approximately 33% higher odds of medical disputes (1.059⁵ ≈ 1.33). In contrast, each one-point increase in perceived healthcare service quality score was associated with approximately 2.0% lower odds of medical disputes, and a 5-point increase corresponded to approximately 9.6% lower odds (0.980⁵ ≈ 0.904).

**Fig. 1:**
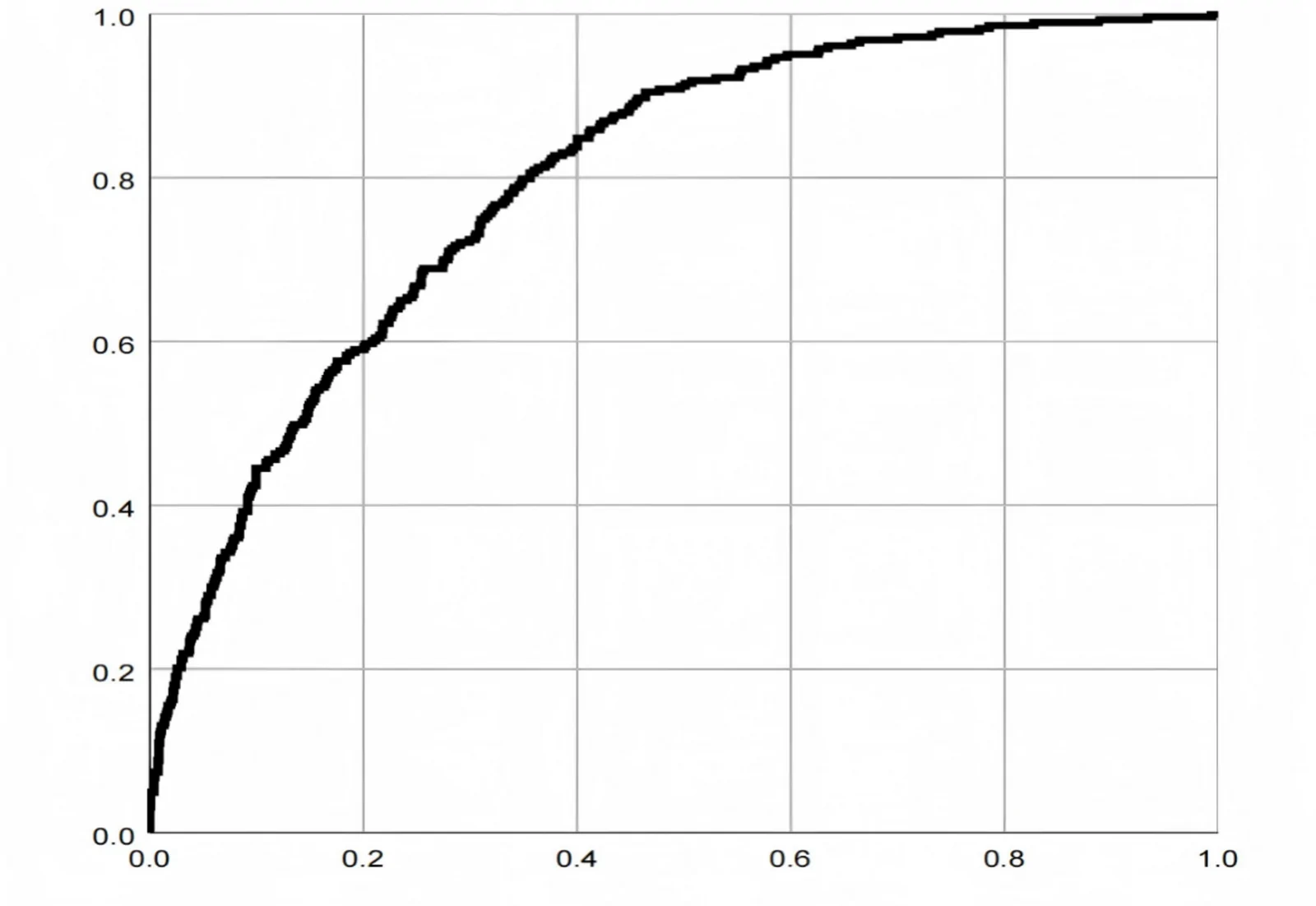
ROC curve of the multivariable logistic regression model for medical disputes

Model diagnostics demonstrated acceptable model performance. The model showed acceptable discriminatory ability, with a C-statistic/area under the receiver operating characteristic curve (AUC) of 0.797 shown in Fig. 1. The Hosmer–Lemeshow goodness-of-fit test indicated satisfactory calibration of the final model (*p* > 0.05).

## Discussion

This cross-sectional study examined factors associated with medical disputes from physicians and medical technicians’ perspectives, with particular attention to occupational burnout and perceived healthcare service quality. Our findings indicate that higher perceived healthcare service quality was associated with lower odds of involvement in medical disputes, whereas occupational burnout, male gender, longer weekly working hours, higher daily patient volume, and turnover intention were associated with higher odds of reporting medical disputes.

Out of the physicians and medical technicians who participated in the survey, approximately 18.0% reported that they had been involved in medical disputes during the past year. The prevalence of medical disputes observed in this study was lower than the 33.48% reported by Wu et al. [29]. One possible explanation is the difference in sampling frameworks. Our study included participants from both tertiary hospitals and community health centers, whereas previous studies have primarily focused on high-volume tertiary hospitals, where clinical complexity and workload are generally greater. This broader institutional representation may partly explain the relatively lower prevalence of medical disputes observed in our study.

Consistent with previous research [30, 31], male participants were found to be more likely to report dispute experiences. Prior evidence suggests that female participants typically employ more collaborative and patient-centered communication styles—characterized by empathy, emotional reassurance, and encouragement of patient participation, which have been associated with higher patient satisfaction and mitigate conflict risk [32]. Conversely, male participants often adopt more task-oriented communication patterns, focusing primarily on diagnostic and therapeutic tasks with less emotional engagement [33]. Within an inherently asymmetric doctor-patient communication context, such an approach may be perceived as distant or insufficiently attentive, thereby heightening dissatisfaction [33]. In addition, some previous studies have suggested that patients may perceive female physicians as more meticulous and communicative, aligning more closely with their psychological expectations [34, 35]. Conversely, when expectations of male physicians regarding technical expertise and professional authority are not fully met, patients may be more likely to attribute unfavorable outcomes to provider-related factors, which has been suggested as a potential contributor to complaints or medical disputes [36].

Aligned with earlier evidence [37], this study also found that longer weekly working hours and greater daily patient volume were associated with dispute involvement. One possible explanation is that heavier workloads place greater time pressure and cognitive demands on physicians and medical technicians [38]. Under these circumstances, healthcare professionals may have fewer opportunities to obtain comprehensive medical histories, provide detailed explanations of diagnoses and treatment plans, or respond fully to patients’ concerns. These constraints may be associated with less effective doctor-patient communication, lower patient satisfaction, and greater potential for misunderstandings or unmet expectations, factors that have previously been linked to medical disputes [39]. In addition, prolonged exposure to heavy workloads has been associated with higher levels of occupational stress and burnout among healthcare professionals [40], which may further influence communication and clinical practice.

The rate of burnout reported in this study was 19.17%, lower than the 31.28% observed in existing research [29]. This difference may partly reflect differences in study populations, as our sample included physicians and medical technicians from both community hospitals and public general hospitals. Compared with healthcare professionals working in large tertiary hospitals, those employed in community hospitals may experience relatively lower patient volumes, less complex clinical cases, and more stable work schedules [41, 42]. These structural characteristics may mitigate sustained emotional exhaustion and professional disengagement, thereby contributing to a lower overall prevalence of burnout at the sample level. In contrast, previous studies reporting higher burnout rates have predominantly focused on physicians in tertiary or high-volume public hospitals, where intense clinical demands and administrative pressures are more pronounced. Importantly, sustained clinical demands, administrative burdens, and cumulative psychological pressure remain salient features of physicians and medical technicians’ work [29]. Previous studies have consistently reported that these occupational demands are associated with fatigue, reduced cognitive performance, diminished interpersonal engagement, and lower professional fulfillment [43].

Importantly, this study found that higher perceived healthcare service quality was associated with lower odds of medical disputes. Although this relationship has received limited attention in previous research, our findings suggest that the way care is organized and delivered may play a role in shaping patients’ perceptions and responses. More structured clinical processes, clearer explanations, and more consistent follow-up procedures could potentially reduce misunderstandings during medical encounters, particularly in high-workload settings. In addition, healthcare environments characterized by higher perceived service quality may support more effective communication between healthcare professionals and patients and their families. Improved transparency and clearer communication during diagnosis and treatment may help alleviate uncertainty and dissatisfaction, which are factors that have been associated with conflict escalation in previous studies [44]. As one of the early studies to explore this association from physicians and medical technicians’ perspectives, our findings provide preliminary evidence that perceived service quality may be relevant to medical dispute occurrence. However, given the cross-sectional design, causality and directionality cannot be established.

In this study, 24.8% of participants expressed turnover intention, which was lower than 39% reported by Wang et.al [45]. We found that participants with turnover intention were more likely to report experiencing medical disputes. One possible explanation is that healthcare workers with higher levels of occupational stress, burnout, or job dissatisfaction may be more vulnerable to communication difficulties, reduced work engagement, and strained DPR, which could increase the likelihood of conflict situations and reported disputes [46]. Conversely, experiencing medical disputes may also be associated with greater psychological distress, emotional exhaustion, and reduced professional satisfaction, which may in turn be related to turnover intention. However, because this study employed a cross-sectional design, the temporal sequence and direction of these associations cannot be determined. The observed relationship may therefore be bidirectional. Future longitudinal studies are needed to clarify the complex relationships among turnover intention, occupational stress, burnout, and medical disputes.

## Strengths and limitations

This study is among the early investigations examining medical disputes from physicians and medical technicians’ perspectives while integrating occupational burnout and perceived healthcare service quality. In addition, participants were recruited from both tertiary hospitals and community health centers, allowing representation of different healthcare settings within the study region. However, several limitations should be acknowledged. First, the cross-sectional design precludes causal inference and limits determination of temporal relationships. Potential reverse causation cannot be excluded, as medical disputes may also influence burnout, psychological status, and turnover intention. Second, several psychosocial variables were assessed using single-item self-reported measures, which may have introduced reporting bias and limited measurement precision. Third, although multiple covariates were adjusted for, residual confounding from unmeasured factors, such as clinical specialty, department type, years of experience, and institutional dispute-management mechanisms, may still exist. Fourth, the study adopted a broad operational definition of medical disputes that included both formal disputes and conflict-related incidents, such as verbal abuse and online harassment. While this may better reflect real-world physician–patient conflicts in China, it may also have contributed to a higher estimated prevalence of dispute experiences compared with studies using narrower legal or administrative definitions. Finally, because the sample was derived from healthcare institutions in Xuzhou, the generalizability of the findings to other regions may be limited. Future longitudinal and multi-center studies are warranted to further validate these findings.

## Conclusion

In this study, approximately 18% of physicians and medical technicians reported experiencing at least one medical dispute in the past year. Higher levels of occupational burnout, longer weekly working hours, and greater daily patient volume were associated with higher odds of reporting medical disputes, whereas higher perceived healthcare service quality was associated with lower odds of reporting medical disputes. These findings highlight several occupational and organizational characteristics that may be relevant to medical dispute experiences among healthcare professionals. Although causal relationships cannot be inferred from this cross-sectional study, the observed associations suggest that initiatives aimed at improving working conditions, reducing occupational burnout, and enhancing healthcare service quality and communication may warrant further investigation in efforts to reduce medical disputes.

## Supplementary Information

Below is the link to the electronic supplementary material.

Supplementary Material 1

## Data Availability

The datasets used and/or analyzed during the current study are available from the corresponding author on reasonable request.

## Abbreviations

DPR: Doctor-patient relationship
CNKI: China National Knowledge Infrastructure
OLBI: Oldenburg Burnout Inventory
EX: Exhaustion
DEM: Disengagement
VIF: Variance inflation factor
CQS: Chirurgisches Qualitätssiegel
SD: Standard Deviation
AUC: Area Under Curve
ROC: Receiver operating characteristic
