# Supplementary material for: Association of occupational burnout and perceived healthcare service quality with medical disputes among physicians and medical technicians: a cross-sectional study in China
Source: BMC Health Serv Res. 2026 Jul 13;26:1368. doi: 10.1186/s12913-026-15142-5 (PMC13647523; doi:10.1186/s12913-026-15142-5)
Supplement: Supplementary file 1 [file 12913_2026_15142_MOESM1_ESM.pdf]

Questionnaire on Physician–Patient Relationship
(English Version)

Introduction

Dear Healthcare Professional,

Thank you for taking the time to participate in this survey. The purpose of this questionnaire is to better understand your experiences, perceptions, and needs regarding physician–patient relationships and medical disputes. Your responses are anonymous and will be used for research purposes only. Please answer all questions honestly.

Part I. Baseline Information

1.Gender
☐ Male
☐ Female

2.Age
☐ 18–30 years
☐ 31–40 years
☐ 41–50 years
☐ 51 years or above

3.Years of practice
☐ < 5 years
☐ 5–10 years
☐ 10–20 years
☐ 20–30 years
☐ ≥ 30 years

4.Marital status
☐ Married
☐ Single (including divorced or widowed)

5.Education level
☐ High school / technical secondary school
☐ Junior college
☐ Bachelor’s degree
☐ Master’s degree or above

6.Professional title
☐ None
☐ Junior
☐ Intermediate

☐ Senior

7.Working hours per week
☐ < 40 hours
☐ 40–55 hours
☐ > 55 hours

8.Daily patient volume
☐ < 20 patients
☐ 20–30 patients
☐ 30–40 patients
☐ > 40 patients

9.Perceived work pressure
☐ High
☐ Moderate
☐ Low

10.Average monthly income
☐ ≤ 5,000 RMB
☐ 5,001–10,000 RMB
☐ 10,001–20,000 RMB
☐ ≥ 20,001 RMB

11.Perceived income level
☐ High
☐ Moderate
☐ Low

12.Employment type
☐ Permanent staff
☐ Contract-based employment
☐ Resident / intern

13.Do you undertake teaching duties (including part-time)?
☐ Yes
☐ No

14.Do you perform administrative duties (including part-time, e.g., department head, nurse manager)?
☐ Yes
☐ No

15.Does your hospital provide medical liability insurance?

☐ Yes
☐ No

16.Have you experienced any medical disputes in the past 12 months?
(including verbal abuse, minor physical altercations, online harassment, personal attacks, obstruction of medical services, etc.)
☐ Yes
☐ No

17.Have you experienced medical violence in the past 12 months?
☐ Yes
☐ No

18.How would you rate your relationship with patients?
☐ Good
☐ Moderate
☐ Poor

19.How would you rate your professional competence?
☐ High
☐ Moderate
☐ Low

20.Self-rated physical health status
☐ Good
☐ Moderate
☐ Poor

21.Self-rated mental health status
☐ Good
☐ Moderate
☐ Poor

22.Your service attitude toward patients
☐ Good
☐ Moderate
☐ Poor

23.Your communication with patients
☐ Good
☐ Moderate
☐ Poor

24.Your working/clinical environment

☐ Good
☐ Moderate
☐ Poor

25.Do you have turnover intention (intention to resign or change profession)?
☐ Yes
☐ No

26.Have you participated in training related to medical disputes or physician–patient relationships?
☐ Yes
☐ No

27.Do you think the hospital's performance evaluation system is reasonable?
☐ Reasonable
☐ Fair
☐ Unreasonable

Part II. Occupational Burnout (Oldenburg Burnout Inventory, OLBI)
Instruction:
Please indicate how strongly you agree with each statement based on your own experience.
1 = Strongly disagree, 2 = Disagree, 3 = Agree, 4 = Strongly agree
（1）Exhaustion
28.I always find new and interesting aspects in my work.
☐ Strongly disagree
☐ Disagree
☐ Agree
☐ Strongly agree

29.There are days when I feel tired before I arrive at work
☐ Strongly disagree
☐ Disagree
☐ Agree
☐ Strongly agree

30.It happens more and more often that I talk about my work in a negative way.
☐ Strongly disagree
☐ Disagree
☐ Agree
☐ Strongly agree

31.After work, I tend to need more time than in the past in order to relax and feel better.

☐ Strongly disagree
☐ Disagree
☐ Agree
☐ Strongly agree

32.I can tolerate the pressure of my work very well.
☐ Strongly disagree
☐ Disagree
☐ Agree
☐ Strongly agree

33.Lately, I tend to think less at work and do my job almost mechanically
☐ Strongly disagree
☐ Disagree
☐ Agree
☐ Strongly agree

34.I find my work to be a positive challenge.
☐ Strongly disagree
☐ Disagree
☐ Agree
☐ Strongly agree

35.During my work, I often feel emotionally drained.
☐ Strongly disagree
☐ Disagree
☐ Agree
☐ Strongly agree

（2）Disengagement
36.Over time, one can become disconnected from this type of work
☐ Strongly disagree
☐ Disagree
☐ Agree
☐ Strongly agree

37.After working, I have enough energy for my leisure activities
☐ Strongly disagree
☐ Disagree
☐ Agree
☐ Strongly agree

38.Sometimes I feel sickened by my work tasks.
☐ Strongly disagree

☐ Disagree
☐ Agree
☐ Strongly agree

39.After my work, I usually feel worn out and weary.
☐ Strongly disagree
☐ Disagree
☐ Agree
☐ Strongly agree

40.This is the only type of work that I can imagine myself doing
☐ Strongly disagree
☐ Disagree
☐ Agree
☐ Strongly agree

41.Usually, I can manage the amount of my work well.
☐ Strongly disagree
☐ Disagree
☐ Agree
☐ Strongly agree

42.I feel more and more engaged in my work.
☐ Strongly disagree
☐ Disagree
☐ Agree
☐ Strongly agree

43.When I work, I usually feel energized.
☐ Strongly disagree
☐ Disagree
☐ Agree
☐ Strongly agree

Part III. Perceived Healthcare Service Quality(Chirurgisches Qualitätssiegel, CQS)

Instruction:
Please indicate the extent to which each statement describes your routine clinical practice.
1 = Very poor, 2 = Poor, 3 = Fair, 4 = Good, 5 = Very good

（1）Psychosocial Care
44.Informing patients in a timely manner about diagnostic and treatment methods and their rationale.

☐ Very poor
☐ Poor
☐ Fair
☐ Good
☐ Very good

45.Showing sympathy and concern for patients and their families.
☐ Very poor
☐ Poor
☐ Fair
☐ Good
☐ Very good

46.Providing necessary emotional support to patients.
☐ Very poor
☐ Poor
☐ Fair
☐ Good
☐ Very good

47.Involving patients in decision-making during the diagnostic and treatment process.
☐ Very poor
☐ Poor
☐ Fair
☐ Good
☐ Very good

48.Taking psychosocial factors related to the disease into consideration.
☐ Very poor
☐ Poor
☐ Fair
☐ Good
☐ Very good

（2）Diagnostic and Therapeutic Procedures
49.Performing surgical procedures in a timely and accurate manner.
☐ Very poor
☐ Poor
☐ Fair
☐ Good
☐ Very good

50.Conducting a comprehensive and accurate assessment of patients' symptoms.
☐ Very poor

☐ Poor
☐ Fair
☐ Good
☐ Very good

51.Making correct and appropriate disease diagnoses.
☐ Very poor
☐ Poor
☐ Fair
☐ Good
☐ Very good

52.Selecting appropriate treatment strategies based on evaluation of medical evidence.
☐ Very poor
☐ Poor
☐ Fair
☐ Good
☐ Very good

（3）Quality Assurance
53.Properly maintaining medical records and ensuring their accuracy and completeness.
☐ Very poor
☐ Poor
☐ Fair
☐ Good
☐ Very good

54.Making optimal use of available medical resources.
☐ Very poor
☐ Poor
☐ Fair
☐ Good
☐ Very good

55.Adhering to medical standards and clinical guidelines during diagnosis and treatment.
☐ Very poor
☐ Poor
☐ Fair
☐ Good
☐ Very good

56.Continuously improving healthcare services to enhance overall service quality.

☐ Very poor
☐ Poor
☐ Fair
☐ Good
☐ Very good
